# Supplementary material for: Prediabetes in adult Saudis: a systematic review & meta-analysis of prevalence studies (2000–2024)
Source: PeerJ. 2025 Aug 20;13:e19778. doi: 10.7717/peerj.19778 (PMC12374689; doi:10.7717/peerj.19778)
Supplement: Supplemental Information 2 [file peerj-13-19778-s002.docx]

**Supplementary file 1**

**Title: Prediabetes in Adult Saudis: A Systematic Review & Meta-Analysis of Prevalence Studies (2000–2024)**

**Abdelmarouf Mohieldein^1*^, Habeeb Ali Baig^2^, Mahmoud Elhabiby^3^, Abdullah Almushawwah^1^, Mohamed Salih Mahfouz^4^, Atif H. Khirelsied^5^**, **Nour Abdelmarouf ^6^**, **Gad Allah Modawe^7^.**

^1^Department of Medical Laboratories, College of Applied Medical Sciences, Qassim University, Buraidah Saudi Arabia.
^2^Department of Microbiology. College of Medicine. Northern Border University, Arar, Saudi Arabia. Email: HABEEB.BAIG@nbu.edu.sa ORCID: https://orcid.org/0000-0002-5093-0623
^3^Department of Medical Laboratory Sciences, Al-Aqsa University, Gaza, Palestine. ORCID: https://orcid.org/0009-0000-9622-2633
^4^Department Family and Community Medicine, Faculty of Medicine, Jazan University, Jazan City, Saudi Arabia. Email: [mmahfouz@jazanu.edu.sa](mailto:mmahfouz@jazanu.edu.sa). ORCID: 0000-0002-3155-6381
^5^Department of Biochemistry. Faculty of Medicine. Al-Baha University. Al Baha city. Saudi Arabia. Email: [akhirelsied@bu.edu.sa](mailto:akhirelsied@bu.edu.sa). ORCID 0009-0005-0213-3493.

^6^student. Faculty of Medicine, Alexandria University, Alexandria, Egypt.

^7^Omdurman Islamic University, Faculty of Medicine and Health Sciences, Department of Biochemistry, Omdurman, Sudan. Email: gadobio77@oiu.edu.sd

*Corresponding author: Prof. Abdelmarouf Mohieldein. Department of Medical Laboratories, College of Applied Medical Sciences, Qassim University. P.O. Box: 6699, Buraidah 51452, Qassim, Saudi Arabia. Email: [fky@qu.edu.sa](mailto:fky@qu.edu.sa). ORCID: <https://orcid.org/0000-0003-2105-3438>

.

**Contents:**

**The metadata supplementary file was organized according to the order in which the contents first appeared in the manuscript.**

- The PRISMA 2020 Abstract Checklist
- Table S.1: Preferred Reporting Items for Systematic Reviews and Meta-analyses (PRISMA) checklist
- Table S. 2: Search strategy for studies published between January 2000 and September 2024
- Table S. 3: Justification for excluding articles during full-text screening (n=30)
- Table S. 4: The quality of the extracted articles using the Joanna Briggs Institute (JBI) quality assessment scale for prevalence studies (JBI, 2020)
- Figure S.1: Sensitivity test for pooled prevalence.
- Figure S. 2: Funnel Plot for publication bias.
- Table S.5: Egger's regression intercept.
- Table S.6: Heterogeneity findings.
- Figure S.3: Forest plot for findings of subgroup analysis according to the gender of the population in samples of selected studies.
- Figure S. 4: Forest plot Sensitivity test of subgroup analysis based on Gender, excluding Al Shehri study.
- Figure S. 5: Forest plot of the pooled prevalence of prediabetes by the regions where the studies were conducted.
- Figure S. 6: Forest plot Sensitivity test of subgroup analysis based on the Central region, excluding Al Shehri study.
- Figure S. 7: Forest plot Sensitivity test of subgroup analysis based on quality assessment of the studies.
- Table S.7: The Grading of Recommendations Assessment, Development and Evaluation (GRADE) summary of the overall certainty of evidence for the pooled prevalence estimate
- Table S.8. A & B. Secondary Outcomes, Aljaadi et al. (2023)
- Table S. 9: Summary of pooled prevalence estimates of prediabetes from various meta-analyses across different populations

**The PRISMA 2020 Abstract Checklist**

| **Section and Topic** | **Item #** | **Checklist item** | **Reported (Yes/No)** |
| --- | --- | --- | --- |
| **TITLE** | | |  |
| Title | 1 | Identify the report as a systematic review. | Yes |
| **BACKGROUND** | | |  |
| Objectives | 2 | Provide an explicit statement of the main objective(s) or question(s) the review addresses. | Yes |
| **METHODS** | | |  |
| Eligibility criteria | 3 | Specify the inclusion and exclusion criteria for the review. | Yes |
| Information sources | 4 | Specify the information sources (e.g. databases, registers) used to identify studies and the date when each was last searched. | Yes |
| Risk of bias | 5 | Specify the methods used to assess risk of bias in the included studies. | Yes |
| Synthesis of results | 6 | Specify the methods used to present and synthesise results. | Yes |
| **RESULTS** | | |  |
| Included studies | 7 | Give the total number of included studies and participants and summarise relevant characteristics of studies. | Yes |
| Synthesis of results | 8 | Present results for main outcomes, preferably indicating the number of included studies and participants for each. If meta-analysis was done, report the summary estimate and confidence/credible interval. If comparing groups, indicate the direction of the effect (i.e. which group is favoured). | Yes |
| **DISCUSSION** | | |  |
| Limitations of evidence | 9 | Provide a brief summary of the limitations of the evidence included in the review (e.g. study risk of bias, inconsistency and imprecision). | Yes |
| Interpretation | 10 | Provide a general interpretation of the results and important implications. | Yes |
| **OTHER** | | |  |
| Funding | 11 | Specify the primary source of funding for the review. | Yes |
| Registration | 12 | Provide the register name and registration number. | Yes |

**Table S.1: Preferred Reporting Items for Systematic Reviews and Meta-analyses (PRISMA) checklist**

| **Section/topic** | **#** | **Checklist item** | **Reported on page #** |
| --- | --- | --- | --- |
| **TITLE** | | |  |
| Title | 1 | Identify the report as a systematic review, meta-analysis, or both. | 1 |
| **ABSTRACT** | | |  |
| Structured summary | 2 | Provide a structured summary including, as applicable: background; objectives; data sources; study eligibility criteria, participants, and interventions; study appraisal and synthesis methods; results; limitations; conclusions and implications of key findings; systematic review registration number. | 3 |
| **INTRODUCTION** | | |  |
| Rationale | 3 | Describe the rationale for the review in the context of what is already known. | 4 |
| Objectives | 4 | Provide an explicit statement of questions being addressed with reference to participants, interventions, comparisons, outcomes, and study design (PICOS). | 4 |
| **METHODS** | | | |
| Protocol and registration | 5 | Indicate if a review protocol exists, if and where it can be accessed (e.g., Web address), and, if available, provide registration information including registration number. | 5 |
| Eligibility criteria | 6 | Specify study characteristics (e.g., PICOS, length of follow-up) and report characteristics (e.g., years considered, language, publication status) used as criteria for eligibility, giving rationale. | 4 |
| Information sources | 7 | Describe all information sources (e.g., databases with dates of coverage, contact with study authors to identify additional studies) in the search and date last searched. | 4&5 |
| Search | 8 | Present full electronic search strategy for at least one database, including any limits used, such that it could be repeated. | Table S.2 |
| Study selection | 9 | State the process for selecting studies (i.e., screening, eligibility, included in systematic review, and, if applicable, included in the meta-analysis). | 5 |
| Data collection process | 10 | Describe method of data extraction from reports (e.g., piloted forms, independently, in duplicate) and any processes for obtaining and confirming data from investigators. | 5 |
| Data items | 11 | List and define all variables for which data were sought (e.g., PICOS, funding sources) and any assumptions and simplifications made. | 5 |
| Risk of bias in individual studies | 12 | Describe methods used for assessing risk of bias of individual studies (including specification of whether this was done at the study or outcome level), and how this information is to be used in any data synthesis. | 6 |
| Summary measures | 13 | State the principal summary measures (e.g., risk ratio, difference in means). | 6 |
| Synthesis of results | 14 | Describe the methods of handling data and combining results of studies, if done, including measures of consistency (e.g., I2) for each meta-analysis. | 6 |
| Risk of bias across studies | 15 | Specify any assessment of risk of bias that may affect the cumulative evidence (e.g., publication bias, selective reporting within studies). | 6 |
| Additional analyses | 16 | Describe methods of additional analyses (e.g., sensitivity or subgroup analyses, meta-regression), if done, indicating which were pre-specified. | 6 |
| **RESULTS** | | | |
| Study selection | 17 | Give numbers of studies screened, assessed for eligibility, and included in the review, with reasons for exclusions at each stage, ideally with a flow diagram. | 6  & Figure. 1  Table S.4 |
| Study characteristics | 18 | For each study, present characteristics for which data were extracted (e.g., study size, PICOS, follow-up period) and provide the citations. | 7  &Tables 1 & S. 3 |
| Risk of bias within studies | 19 | Present data on risk of bias of each study and, if available, any outcome level assessment (see item 12). | 7 & Figure S.2 |
| Results of individual studies | 20 | For all outcomes considered (benefits or harms), present, for each study: (a) simple summary data for each intervention group (b) effect estimates and confidence intervals, ideally with a forest plot. | 7 & Figure 2 |
| Synthesis of results | 21 | Present results of each meta-analysis done, including confidence intervals and measures of consistency. | 7 & Figure 2 |
| Risk of bias across studies | 22 | Present results of any assessment of risk of bias across studies (see Item 15). | 7 & Figure S. 2 |
| Additional analysis | 23 | Give results of additional analyses, if done (e.g., sensitivity or subgroup analyses, meta-regression [see Item 16]). | 7 - 8 & Figures 3, 4, S.3, S.4, S.5, S.6, S.7, S.8 |
| **DISCUSSION** | | | |
| Summary of evidence | 24 | Summarize the main findings including the strength of evidence for each main outcome; consider their relevance to key groups (e.g., healthcare providers, users, and policy makers). | 8-11 |
| Limitations | 25 | Discuss limitations at study and outcome level (e.g., risk of bias), and at the review-level (e.g., incomplete retrieval of identified research, reporting bias). | 12 |
| Conclusions | 26 | Provide a general interpretation of the results in the context of other evidence, and implications for future research. | 12 |
| **FUNDING** | | | |
| Funding | 27 | Describe sources of funding for the systematic review and other support (e.g., supply of data); role of funders for the systematic review. | 12 |

**Table** **S. 2: Search strategy for studies published between January 2000 and September 2024**

| **String** | **Keywords** |
| --- | --- |
| #1 | ("Saudi Arabia" OR "Kingdom of Saudi Arabia" OR "KSA" OR "Middle East" OR "Arabian Peninsula") |
| #2 | ("Prediabetes" OR "Pre-diabetes" OR "Pre-diabetic" OR "Impaired glucose tolerance" OR "Impaired fasting glucose" OR "Glucose intolerance" |
| #3 | ("Prevalence" OR "Cross-sectional" OR " Epidemiology" OR "Population-based" |
| #4 | ("2000-2024" OR "2000 to 2024" OR "21st century") |
| #5 | ("Fasting plasma glucose" OR "Oral glucose tolerance test" OR "HbA1c" OR "American Diabetes Association" OR "ADA" OR "World Health Organization" OR "WHO") |
| #6 | #1 AND #2 AND #3 AND #4 AND #5 |

**Table** **S. 3: Justification for excluding articles during full-text screening (n=30)**

|  | **Author, Publication year** | **Doi** | **Region of study** | **Region** | **Reason for exclusion** | **Category of reasons for exclusion** |
| --- | --- | --- | --- | --- | --- | --- |
| 1 | Alshaikhi et al., 2024. | 10.2144/fsoa-2023-0208 | Alqunfudah, | Southern | The study included both Saudi and non-Saudi participants. | Population issues |
| 2 | Meo et al., 2020 | Doi:10.3390/ijerph17113992 | Riyadh | Central | The study did not clearly specify Saudi population  The study focused exclusively on male workers from specific industries, which does not represent the general adult Saudi population. | Population issues |
| 3 | Bahijri et al., 2016. | 10.1371/journal.pone.0152559 | Jeddah | Western | The study included both Saudi and non-Saudi participants. | Population issues |
| 4 | Meo et al., 2020 | Https://doi.org/10.12669/pjms.36.2.1266 | Riyadh | Central | The study did not clearly specify Saudi population  The study focused exclusively on male workers from cement industry, which does not represent the general adult Saudi population. | Population issues |
| 5 | Meo et al., 2021 | Https://doi.org/10.12669/pjms.37.4.4128 | Riyadh | Central | The study did not clearly specify Saudi population  The study focused exclusively on male cricket players, which does not represent the general adult Saudi population. | Population issues |
| 6 | Meo et al., 2018 | Https://doi.org/10.1177/1557988318800203 | Riyadh | Central | The study did not clearly specify Saudi population  The study focused exclusively on male workers in the plastic industry, which does not represent the general adult Saudi population. The study participants were primarily workers from the Indian subcontinent, rather than Saudi nationals. | Population issues |
| 7 | Meo et al., 2021 | Https://doi.org/10.3390/ijerph18041763 | Riyadh | Central | The study did not clearly specify Saudi population,  The study focused specifically on male football players | Population issues |
| 8 | Wahabi H., 2018. | Https://doi.org/10.1155/2018/4282347 | Riyadh | Central | The study focused exclusively on women with prior gestational diabetes mellitus (GDM), a specific subgroup of the population. | Population issues |
| 9 | Al-daghri et al., 2011 | Https://doi.org/10.1186/1741-7015-9-76 | Riyadh | Central | The study included individuals ranging from 7 to 80 years of age | Population issues |
| 10 | Alhazmi et al., 2017 | Http://dx.doi.org/10.19082/5531 | Turaif, arar, | Northern | The study included individuals aged between 6 and 63 years | Population issues |
| 11 | Al-khlaiwi.et al., 2022 | *Https://doi.org/10.12669/pjms.38.7.6189* | Riyadh | Central | The study did not specify the Saudi population | Population issues |
| 12 | Al Osaimi et al., 2007 | *---* | Riyadh | Central | The study did not specify the Saudi population | Population issues |
| 13 | Al-Rubeaan et al., 2014. | 10.5144/0256-4947.2014.465 | Nationwide, | Nationwide, | The study included participants ranging from 0 to 100 years of age, | Population issues |
| 14 | El Bcheraoui et al., 2014 | 10.1007/s00038-014-0612-4 | Nationwide, | Nationwide, | The study included Saudis aged 15 years or older | Population issues |
| 15 | Nour Eldein et al et al., 2024 | 10.7759/cureus.57608 | Makkah | Western | The study design was a case-control study | Study design |
| 16 | Amer et al., 2020 | 10.3390/nu12030804 | Riyadh | Central | This study was a randomized controlled trial (RCT) | Study design: |
| 17 | Wahab et al., 2019. | Https://doi.org/10.1371/journal.pone.0210024 | Riyadh | Central | Longitudinal cohort study  Reported incidence of prediabetes on postpartum women | Study design |
| 18 | Elkhateeb et al., 2018. | 10.11648/j.ijbse.20180603.11 | Hail | Northern | The diagnostic criteria for prediabetes were not clearly specified. The study measured blood glucose levels using a glucometer but did not mention standard diagnostic thresholds. | Diagnostic methods |
| 19 | Alshaikh et al., 2024 | Https://doi.org/10.3390/  Medicina60050775 | Abha, aseer | Southern | The study used the Australian Type 2 Diabetes Risk Assessment Tool (AUSDRISK) tool, which is a non-invasive diabetes risk assessment method rather than relying on direct diagnostic methods. | Diagnostic methods |
| 20 | Al-Zamil et al., 2021. | 10.15640/ijhs.v9n1a6 | Riyadh, | Central | The study used random blood glucose testing to assess prediabetes. | Diagnostic methods |
| 21 | Al-Baghli et al., 2010. | --- | Dammam, | Eastern | The used diagnostic methods include capillary fasting blood glucose (CFBG) and casual capillary blood glucose (CCBG) as screening then only positive results were confirmed measurement of fasting plasma glucose levels from a venous sample | Diagnostic methods |
| 22 | Alghamdi et al., 2013. | Https://doi.org/10.1097/maj.0b013e318287c96c | Jeddah | Western | The study initially used random blood glucose levels (RBGLS) followed by oral glucose tolerance tests (OGTT) for those only have RBGLS ≥110 mg/d to identify prediabetes. | Diagnostic methods |
| 23 | Abdelrahman A S et al., 2018 | 10.5539/gjhs.v10n3p161 | Rabigh | Western | The study did not mention of employing ADA or WHO criteria rather glucose-tolerance was tested using fast gluco-test (Bayer contour instrument with blood glucose test strips). | Diagnostic methods |
| 24 | Al Shuwaysh et al., 2023 | 10.7759/cureus.41926 | Al-ahsa | Eastern | The study did not mention of employing ADA or WHO accepted diagnostic criteria but instead it applied the CDC Prediabetes Risk Test. | Diagnostic methods |
| 25 | Afifi et al., 2015 | 10.1007/s13410-013-0189-0 | Taif | Western | The study utilized a random blood sugar (RBS) screening test with a threshold of ≥200 mg/dl as the sole method for diagnosing prediabetes. | Diagnostic methods |
| 26 | Afifi et al., 2017 | 10.4236/jdm.2017.71002 | Wadi al-dawasir | Central | The study used random plasma glucose (RPG) as the primary diagnostic tool with a cutoff of ≥200 mg/dl for identifying prediabetes. | Diagnostic methods |
| 27 | Hobani et al. (2015) | 10.3126/ijasbt.v3i4.13921 | Jazan | Southern | Study considered the 100 -126 mg/d as impaired fasting glucose or prediabetes, thought the ADA criteria is 100 mg/dl – 125 mg/dl Also, study did not report fasting blood collection. These may have its impact of 70% prevalence of prediabetes which seems to be outlier compared to all included studies. | Diagnostic methods |
| 28 | Alreshidi et al., 2023 | Https://doi.org/10.26355/eurrev_202304_32127 | Hail | Northern | The study did not directly specify the prediabetes prevalence form; instead, it reported that all participants had hba1c levels above 6.5% which considered diabetic according to the ADA criteria. | Outcome |
| 29 | Memish et al., 2015 | [10.1089/dia.2014.0267](https://doi.org/10.1089/dia.2014.0267) | Riyadh, | Central | The study developed and validated a noninvasive screening test | outcome |
| 30 | Basheikh et al., 2016. | 10.5455/ijmsph.2016.22012016333 | Jeddah | Western | The study relied on retrospective data extracted from medical files | Data collection methods |

**Table** **S. 4:** The quality of the extracted articles using the Joanna Briggs Institute (JBI) quality assessment scale for prevalence studies (JBI, 2020) (Munn et al., 2015)

| **No.** | **Author** | **Publication year** | **Q1** | **Q2** | **Q3** | **Q4** | **Q5** | **Q6** | **Q7** | **Q8** | **Q9** | **Total score**  **Out of 9** | **Quality Assessment** |
| --- | --- | --- | --- | --- | --- | --- | --- | --- | --- | --- | --- | --- | --- |
| 1 | Abu-Almakarem et al | 2024 | Y | U | Y | Y | Y | Y | Y | Y | U | 7 | Medium |
| 2 | Amri et al. | 2019 | Y | Y | Y | Y | Y | Y | Y | Y | U | 8 | High |
| 3 | Al Shehri et al. | 2022 | Y | Y | Y | Y | Y | Y | Y | Y | U | 8 | High |
| 4 | Aldossari et al. | 2018 | Y | Y | Y | Y | Y | Y | Y | Y | Y | 9 | High |
| 5 | Aldossari et al. | 2020 | Y | Y | Y | Y | Y | Y | Y | Y | Y | 9 | High |
| 6 | Al-Ghamdi et al. | 2018 | Y | Y | Y | Y | Y | Y | Y | Y | U | 8 | High |
| 7 | Alhomaid et al. | 2024 | Y | Y | Y | Y | Y | Y | Y | Y | U | 8 | High |
| 8 | Aljabri et al. | 2018 | Y | Y | Y | Y | Y | Y | Y | Y | U | 8 | High |
| 9 | Alomari et al. | 2022 | Y | Y | Y | Y | Y | Y | Y | Y | Y | 9 | High |
| 10 | Al-Rubeaan et al. | 2015 | Y | Y | Y | Y | Y | Y | Y | Y | Y | 9 | High |
| 11 | Al-Zahrani et al. | 2019 | Y | Y | Y | Y | Y | Y | Y | Y | N | 8 | High |
| 12 | Bahijri et al. | 2020 | Y | Y | Y | Y | Y | Y | Y | Y | N | 8 | High |
| 13 | Fayed et al. | 2022 | Y | Y | Y | Y | Y | Y | Y | Y | N | 8 | High |
| 14 | Latif et al. | 2020 | Y | Y | Y | Y | Y | Y | Y | Y | N | 8 | High |
| 15 | Mirza et al. | 2013 | Y | Y | Y | Y | Y | Y | Y | Y | U | 8 | High |
| 16 | Turki et al. | 2016 | Y | Y | Y | Y | Y | Y | Y | Y | U | 8 | High |
| 17 | Ghoraba et al., | 2016 | N | N | Y | Y | Y | Y | Y | Y | U | 7 | Medium |
| 18 | Al-Nozha et al. | 2004 | Y | Y | Y | Y | Y | Y | Y | Y | Y | 9 | High |
| Average | | | | | | | | | | | | 8.2 |  |

Abbreviations: Y, yes; N, no; U, unclear

**Figure S.1**. Sensitivity test for pooled prevalence. The sensitivity analysis shows that the overall findings are quite resilient (24.1%). The pooled prevalence does not change with the removal of any one study, indicating that the overall estimate is reliable.

**Figure S. 2**. Funnel Plot for publication bias. The studies were spread across a wide range of effect sizes (logit event rates) and standard errors. This was attributed to the different sample sizes of the selected studies.

Table S.5. Egger's regression intercept

Table S.6. Heterogeneity findings

**Figure S.3**. Forest plot for findings of subgroup analysis according to the gender of the population in samples of selected studies

**Figure S. 4:** Forest plot Sensitivity test of subgroup analysis based on Gender, excluding Al Shehri study

**Figure S. 5.** Forest plot of the pooled prevalence of prediabetes by the regions where the studies were conducted

**Figure S. 6:** Forest plot Sensitivity test of subgroup analysis based on the Central region, excluding Al Shehri study

**Figure S. 7:** Forest plot Sensitivity test of subgroup analysis based on quality assessment of the studies.

Table S.7: The Grading of Recommendations Assessment, Development and Evaluation (GRADE) summary of the overall certainty of evidence for the pooled prevalence estimate

| **Domain** | **Assessment** | **Justification** |
| --- | --- | --- |
| Risk of Bias | No serious concerns | The majority of studies (16 out of 18) were of high quality (JBI criteria) |
| Inconsistency | Serious concerns | High heterogeneity (I² = 99.1%) |
| Indirectness | No concerns | The included studies entirely matched the PICO question. |
| Imprecision | No serious concerns | The sample size was 47,718 adult Saudis from the general population  95% confidence interval (CI): 19.5% to 29.4% |
| Publication Bias | No concerns | Funnel plot and Egger’s test showed no bias |
| Overall | Moderate | The certainty of evidence was rated as moderate, with a downgrade for high heterogeneity. No serious concerns were found in the other domains |

**Table S. 8. A.** Secondary Outcomes, Aljaadi et al. (2023)

**Table S. 8. B.** Secondary Outcomes, Aljaadi et al. (2023)

**Table S. 9**. Summary of pooled prevalence estimates of prediabetes from various meta-analyses across different populations

| Studies (ref no.) | Country, population | The total number of studies included | Covered period of meta-analysis | Sample size | Diagnosis criteria | Pooled prevalence of prediabetes |
| --- | --- | --- | --- | --- | --- | --- |
| Present study | Saudi Arabia, Saudi | 18 | 2000-2024 | 47,718 | WHO/ ADA criteria | 24.1% (95% CI: 19.5% to 29.4%) |
| Mirahmadizadeh A et al., (Mirahmadizadeh et al., 2020) | Eastern Mediterranean Region (EMRO) | 48 | 2000 -2018 | 567025 | -- | 12.78% (CI: 10.67-14.89) |
| Bigna et al., (Bigna et al., 2018) | Cameron, Cameroonian | 4 | 2000 - 2017 | 5,872 | WHO definitions | 7.1% (95%CI: 3.0-21.9 |
| Akhtar et al., (Akhtar et al., 2020) | Bangladesh, Bangladeshi | 17 | 1995 - 2019 | 56 452 | -- | 10.1% (95% CI: 6.7–14.0). |
| Akhtar et al., (Akhtar et al., 2022) | Malaysia, Malaysian | 9 | 1995-2021 | 88702 | --- | 11.62% (95% CI, 7.17%–  16.97%,) |
| Asmelash et al., (Asmelash et al., 2023) | East African population (six countries: Uganda, Tanzania, Ethiopia, Kenya, Rwanda, and Sudan) | 21 | 2013 - 2022. | 43,379​ | WHO/ ADA criteria | 12.58 % (95 % CI:10.30, 14.86 %) |
| Yitbarek et al.,(Yitbarek et al., 2021) | Ethiopia, Ethiopian | 9 | 2013 - 2021 | 7664 | WHO | 8.94%, 95% CI (2.60–15.28%), |
